# Encapsulated Control: Shaping Insulin Fibrillation through Polymer Confinement

**DOI:** 10.1021/acs.biomac.5c02110

**Published:** 2025-12-24

**Authors:** Anastasiia Murmiliuk, Sergey K. Filippov, Hiroki Iwase, Kuno Schwärzer, Jürgen Allgaier, Aurel Radulescu

**Affiliations:** † Department of Medical Biotechnology and Translational Medicine, 9304 Università degli Studi di Milano, Segrate 20054, Italy; ‡ Jülich Centre for Neutron Science (JCNS) at Heinz Maier-Leibnitz Zentrum (MLZ), 28334 Forschungszentrum Jülich GmbH, Garching 85747, Germany; § 133859 DWI − Leibniz Institute for Interactive Materials, Aachen 52074, Germany; ∥ Neutron Science and Technology Center, Comprehensive Research Organization for Science and Society (CROSS), Tokai, Ibaraki 319-1106, Japan; ⊥ Jülich Centre for Neutron Science (JCNS-1), Forschungszentrum Jülich, Jülich 52425, Germany

## Abstract

The understanding of insulin conformational changes trapped inside a polymeric capsule is obscure, especially at elevated temperatures above 40 °C. We studied the conformational changes of insulin in bulk solution and upon encapsulation into polymeric self-assemblies formed by poly­(ethylene oxide)-*block*-poly­(*N*,*N*,*N*-trimethylammonioethyl methacrylate) copolymer that is oppositely charged to the protein. We demonstrated that loading insulin into the nanoparticles does not affect its secondary structure but alters the pH sensitivity of insulin, making it more resistant to pH variation in the presence of the polymer. However, the temperature resistance of insulin is weakened in the environment of polyelectrolyte, which causes a lowering of the unfolding temperature, and the conformational changes begin already at 40 °C in the nanoparticle core. For the first time, we report that insulin fibrillation follows distinct pathways in free and encapsulated forms, a difference driven by insulin oligomeric state (hexamer in bulk and trimer within the polyelectrolyte/insulin complex).

## Graphical abstract

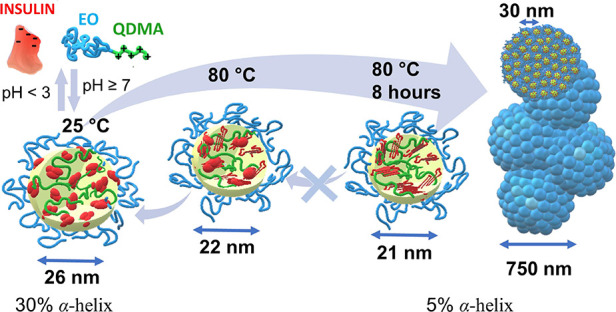

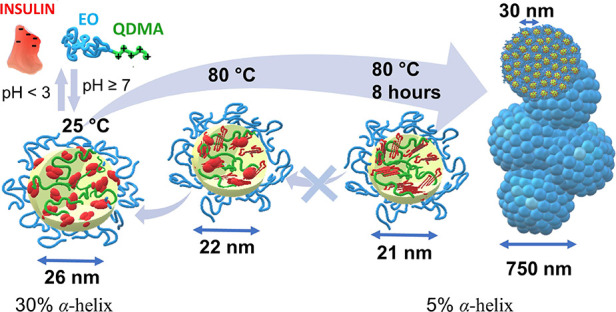

## Introduction

Amyloid fibrils are protein self-assemblies that are naturally formed in living organisms as a result of protein misfolding associated with diseases (Alzheimer’s, type 2 diabetes, and the spongiform encephalopathies) and produced by microorganisms for structure, adhesion, and defense properties.^1,2^ Amyloids are formed by unfolded proteins due to the interaction between exposed hydrophobic segments of the polypeptide backbone that trigger protein aggregation. The formed fibrils can have high stiffness, and therefore, their study is important not only in medical research but also for designing biological nanomaterials with tunable properties from functional amyloids.^3,4^ The needlelike, unbranched morphology of amyloid fibrils arises from laterally bundled protofilaments, each a few nanometers wide and around a micrometer long, with β-strands stacked perpendicularly in a cross-β structure.^3^ The amyloid structure in core regions, secondary structure, protofilament arrangements, and morphology of amyloid depend on the growth conditions, in particular, solvent, temperature, concentration, and agitation.^3,5^

Insulin is widely used in diabetes treatment; however, due to the physicochemical stresses encountered during insulin formulation, storage, delivery, and transport, different types of insulin aggregation may occur, in particular, amyloids that are toxic to the human body.^6,7^ The denaturation of the insulin has been demonstrated to occur in the temperature range between 60 and 80 °C.^8^ However, the unfolding process starts already at 37 °C, even though it has been considered insignificant (1.5% of unfolding). Many environmental factors can affect the unfolding temperature, such as acidity, ionic strength, presence of other proteins, and charged molecules. It has been shown by Vestergaard and coworkers^9,10^ that nuclei of insulin fibrillation are protein oligomers that also elongate the fibrils. They were able to reveal the kinetics of the fibrillation process and describe the 3D structure of insulin at each stage using small-angle X-ray scattering, and hypothesized that the process could be weakened by affecting the oligomer properties. In our previous work, we have demonstrated that insulin exists as a hexamer at physiological pH, while the pH variation and encapsulation into polymer nanoparticles trigger insulin to form trimers and dimers.^11^ Enzymatic degradation of insulin, as well as improved stability, could be achieved by integrating insulin into nanocarriers. Moreover, insulin could be controllably loaded into polymeric nanoparticles and released upon salt or pH variation,^11–14^ and such carriers can be potentially used for the slow insulin release. In addition, the insulin encapsulation into polymeric nanoparticles can enhance its *in vitro* and *in vivo* half-life.^15,16^ The efficacy of insulin encapsulation and preservation of bioactivity after the release of protein has been demonstrated in a number of papers.^17,18^ However, the destabilization of encapsulated protein triggered by various factors and the effect of protein loading into different environments were not thoroughly investigated so far, especially for temperatures above 37 °C. It is widely acknowledged that insulin should be stored at 2–8 °C since it starts aggregating and changing its conformation above this threshold.^19^ Most of the reported research on the properties of polymer-encapsulated insulin or glucagon focused on the elevated temperature range up to the physiological temperature of 37 °C.^20^ However, the understanding of insulin transformation in free and encapsulated states at a higher temperature range between 40–80 °C is of paramount importance since it can provide valuable information on different aspects of fibrillation with and without a polymeric matrix. This consideration is particularly critical for the industrial-scale storage of insulin formulations since high temperatures may change the pathway of insulin fibrillation and, therefore, its therapeutic effect.

In our previous work,^11^ we have shown that human insulin exists mainly in the form of hexamers at pH 7.5, hexamers and trimers at pH 9, and trimers and dimers at pH 3. In addition, we have observed that the encapsulation of insulin at physiological pH is driven by electrostatic interaction with a block copolymer bearing a polycationic (poly­(*N*,*N*-dimethylaminoethyl methacrylate), DMA, and poly­(*N*,*N*,*N*-trimethylammonioethyl methacrylate), QDMA) and a neutral hydrophilic block (poly­(ethylene oxide), EO). The formed core/shell nanoparticles with the core of the polyelectrolyte/insulin complex and the outer shell of EO block can be reversibly disassembled by varying pH. Moreover, the insulin arrangement within the core is governed by salt concentration. However, it is still unclear how pH and temperature variation affect the biological activity and stability of encapsulated insulin at elevated temperatures.

In this work, we investigated the secondary structure and morphology of insulin in bulk solution and insulin loaded into polymer nanoparticles to characterize the protein stability and responsiveness to pH, extended temperature range 25–80 °C, and incubation time. The nanoparticles were formed by electrostatic interaction of positively charged poly­(ethylene oxide)-*block*-poly­(*N*,*N*,*N*-trimethylammonioethyl methacrylate), EO-QDMA, and insulin, which is negatively charged at pH 7.5. EO-QDMA is a block copolymer that can be prepared by simple modification of commercially available poly­(ethylene oxide)-*block*-poly­(*N*,*N*-dimethylaminoethyl methacrylate), EO–DMA, which allows us to decrease the total cost of formulation in comparison to tailor-made polymers. In addition, both blocks of the copolymer are biocompatible, and the polymer ionization degree could be controlled by the quaternization degree of the QDMA block. We used circular dichroism spectroscopy (CD) to determine the secondary structure of insulin with and without polymer, small-angle neutron scattering (SANS) with H_2_O/D_2_O contrast variation to define the morphology of the protein multimers and aggregates before and after fibrillation, and scanning electron microscopy (SEM) to verify the microstructure of the fibrils.

## Materials

Human insulin was purchased from Sigma-Aldrich. Poly­(ethylene oxide)-*block*-poly­(*N*,*N*,*N*-triethylammoniomethyl methacrylate) EO_205_-QDMA_40_ with fully deuterated QDMA block was synthesized by modification of poly­(ethylene oxide)-*block*-poly­(N,N-dimethylaminoethyl methacrylate) with methyl iodide; the synthesis and polymer characterization were reported elsewhere.^11^ Tris­(hydroxymethyl)­aminomethane (Tris), NaCl, NaOH, HCl, NaOD, DCl, and D_2_O were purchased from Sigma-Aldrich and used without further purification.

Insulin was dissolved in 50 mM NaCl and 13 mM Tris solution; a small amount of sodium hydroxide was added to the solution until the insulin was fully dissolved, after which the pH was adjusted to 7.5. EO-QDMA was dissolved in 50 mM NaCl and 13 mM Tris buffer with pH 7.5; no pH adjustment was needed because the polymer is a strong polyelectrolyte. Stock solutions of polymer and insulin were mixed in different ratios, and the polymer concentration was adjusted to 5 g/L.

## Methods

### Small-Angle Neutron Scattering (SANS)

SANS was measured using TAIKAN on the BL15 beamline at the Materials and Life Science Experimental Facility (MLF) in the Japan Proton Accelerator Research Complex (J-PARC).^21^ The neutron wavelength was set to 0.8–7.8 Å. The primary sample-to-detector distance was 5.65 m. The instrument also features other detector banks at shorter distances (3.5, 1.2, and 0.6 m) to perform measurements in a large scattering angle range within a single exposure. The samples were analyzed in a quartz cuvette of 1.0 mm thickness. After applying the corrections for the empty cuvette and dark current contributions, the intensity of the scattering profile was converted to absolute intensity using a standard glassy carbon sample^22^ and radially averaged to obtain the one-dimensional scattering cross section d*S*/d*W* in cm^–1^. The buffer contribution was subsequently subtracted to obtain the scattering of the component of interest. The measurements were performed for insulin and EO-QDMA in D_2_O/H_2_O mixtures of 100/0, 70/30, and 35/65 at 25 °C, after incubation at 80 °C for 8 h, and after cooling to 25 °C. The EO-QDMA/insulin complexes in 100% D_2_O were also scanned by temperature for heating from 25 to 80 °C and cooling from 80 to 25 °C with a rate of 1 °C/min and continuous data collection averaged over a 10 °C temperature interval.

EO-QDMA concentration was 5 g/L, and insulin concentration was 20 g/L. EO-QDMA/insulin in 100% D_2_O was measured during heating and cooling. Samples were heated at 80 °C for 8 h under 3 different contrast conditions (100, 70, and 35% D_2_O) to mask the scattering from either the QDMA block or insulin. Solvent composition required to match scattering from QDMA and insulin was estimated from their theoretical scattering length densities, calculated using the SASfit software.^23^ The SANS curves of EO-QDMA/insulin were fitted using the form factors of Spherical Core with Gaussian Chains Attached,^24,25^ Broad Peak,^26^ and Modified Caillé ^27,28^ or Mass fractal structure factors^29,30^ implemented in the SASfit software.^23^

### Circular Dichroism (CD)

The CD measurements were performed using a Jasco J1100 spectrophotometer in the wavelength range of 180–500 nm using a 1 mm quartz cuvette. For all samples, the insulin concentration was 0.2 g/L, chosen from the concentration series measurement for optimal absorbance range. The EO-QDMA concentrations were 0.05 and 0.2 g/L. The curves were fitted using the BestSel web platform for secondary structure determination and fold recognition.^31^ All fitting curves, details of the fitting model, and fitting parameters are presented in the Supporting Information (Figures S1–S5). Prior to fitting, the measured ellipticity (in mdeg) was converted into molar extinction, Δ*ε* (in M^–1^ cm^–1^) for a 1 mm quartz cuvette, protein concentration of 34 μM, and 51 residues per protein molecule. The experimental curve was smoothed by data averaging on a 2 nm window.

### Scanning Electron Microscopy (SEM)

SEM experiments were performed using a Hitachi S-4800 microscope using the secondary electron detector at different angles. 10 μL of insulin or EO-QDMA/insulin solutions was applied on a grid (Carbon Film 200 Mesh Copper Grids, Electron Microscopy Sciences) and dried at room temperature.

## Results and Discussion

### Impact of Insulin Encapsulation on Protein Structure

In our previous research,^11^ we have shown that insulin forms complexes with tthe polycationic EO-QDMA polymer at a pH above 7. At this pH value, insulin (isoelectric point, p*I*, is 5.2)^11^ is negatively charged, and therefore the complex formation is driven by electrostatic attraction to the strong polyelectrolyte EO-QDMA, and the complex remains stable at higher pH (up to 11) but dissociates at pH below the isoelectric point of insulin. In addition, using small-angle X-ray scattering (SAXS), size exclusion chromatography (SEC), and dynamic light scattering (DLS), we have observed earlier that insulin forms oligomers of different sizes in bulk and in the complex, depending on pH. The insulin hexamers are formed at pH 7.5 and rearrange to trimers after encapsulation, which has been confirmed by the shift in equilibrium between hexamers and trimers after insulin release at pH 9 from SEC measurement; 1.5 times more trimers are present in the solution at pH 9 after insulin release from the complexes in comparison to insulin in the absence of polymer. This rearrangement might affect the conformation of insulin within the complex and its biological activity. In this work, we focus on the insulin fibrillation processes and the understanding of insulin secondary structure at different pH and within polymer nanoparticles, which is crucial for this research. To validate this effect, we performed circular dichroism (CD) experiments for insulin in bulk and encapsulated into the complex with EO-QDMA. In contrast to insulin, polymers are not optically active (see Figure S1 in the Supporting Information), and therefore, only the signal from the insulin is detected.

Figure 1 shows the CD spectra of insulin in bulk and encapsulated into the complexes with EO-QDMA. The presence of a positive maximum at 198 nm and 2 negative minima at 209 and 223 nm indicates that the proteins have predominantly α-helical structures for insulin in bulk and after encapsulation. The encapsulation of insulin does not significantly affect the CD profiles in the wavelength range from 200 to 250 nm. However, at high polymer concentration, the intensity of the maximum at 198 nm decreases by 28%. In order to quantitatively characterize secondary structure changes in insulin conformation, we used the BestSel web server^31,32^ that allows us to fit CD curves and calculate the fractions of secondary structures basis components, including α-helices, parallel and antiparallel β-sheets, and turns. Fitting the CD profiles using the BestSel showed that increasing polymer concentration leads to a slight decrease of helix fraction (from 37 to 34%), appearance of parallel beta sheets with a fraction of 7% (Figure 1, right), and a slight increase in the fraction of turns (from 12 to 15%) that indicates more compact conformation of insulin in the core of the nanoparticle.

**Figure 1:**
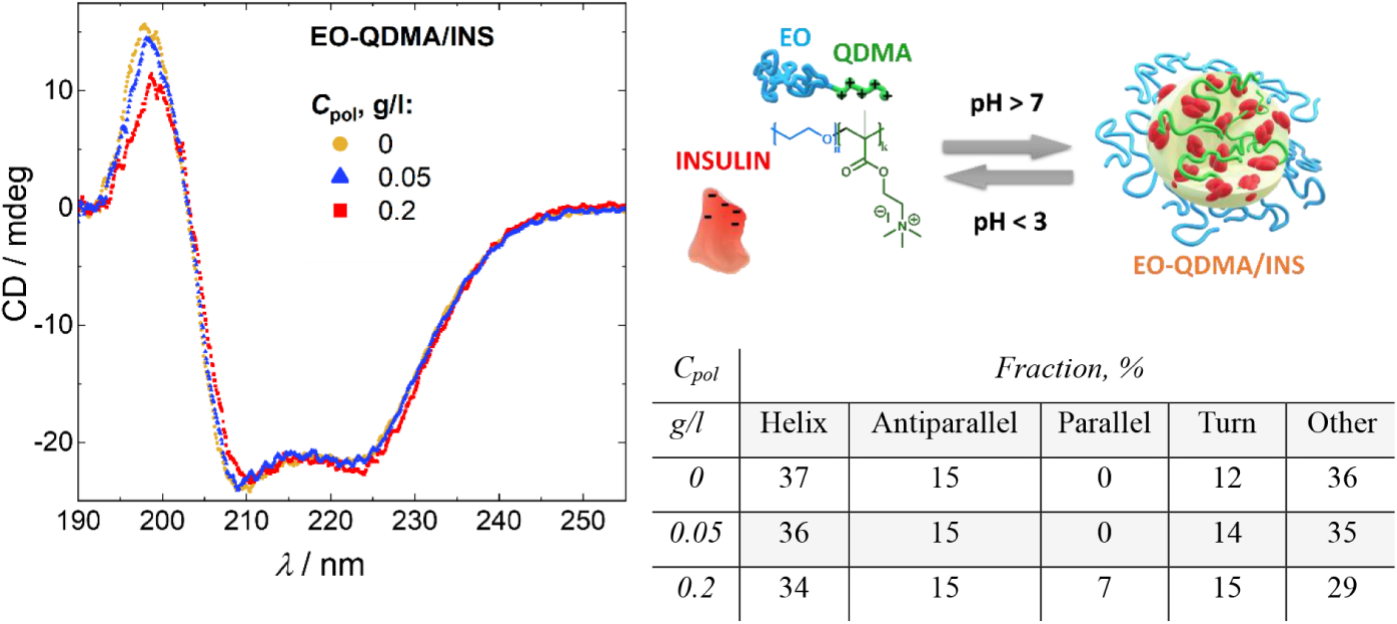
(Left) CD spectra of insulin and EO-QDMA/insulin complexes at pH 7.5 and polymer concentrations 0, 0.05, and 0.2 g/L. (Right) Schematic representation of the studied system: poly­(ethylene oxide)-*block*-poly­(*N*,*N*,*N*-dimethylammonioethyl methacrylate) (EO-QDMA) and insulin form core/shell particles with the EO shell and the core composed of QDMA block and ordered insulin trimers. The particles are formed at pH above 7 and are disrupted at pH <3.^11^ Table with the fractions of α-helices, parallel and antiparallel β-sheets, turns, and other secondary structure basis components obtained from fitting CD spectra using the BestSel method.^31^

The pH sensitivity of insulin was also verified using CD spectroscopy (Figure 2). At pH 3 and 7.5, the CD spectra have similar profiles, indicating that the transition via isoelectric point (p*I* from potentiometric titration is 5.2)^11^ and charge inversion do not affect the conformation of the protein significantly. On the contrary, the increase of pH leads to a 5-fold decrease in the intensity of the maxima at 198 nm and a 1.4 times decrease in the intensity of the minima at 223 nm, indicating the formation of β-sheets. Similar effects are observed for CD profiles of the insulin encapsulated into EO-QDMA complexes (see Figure S2); however, the higher the polymer concentration, the weaker the effect is. The fitting of CD spectra confirms the decrease in the fraction of α-helices for insulin in bulk at high pH in comparison to physiological pH by 7 percentage points, while for the encapsulated insulin, it decreases by 6 percentage points for low polymer content and 0.4 percentage points for higher polymer concentration (Figure 2, right). This observation indicated that insulin encapsulation preserves the protein from conformational change under high pH, and insulin is more stable in a polyelectrolyte environment.

**Figure 2:**
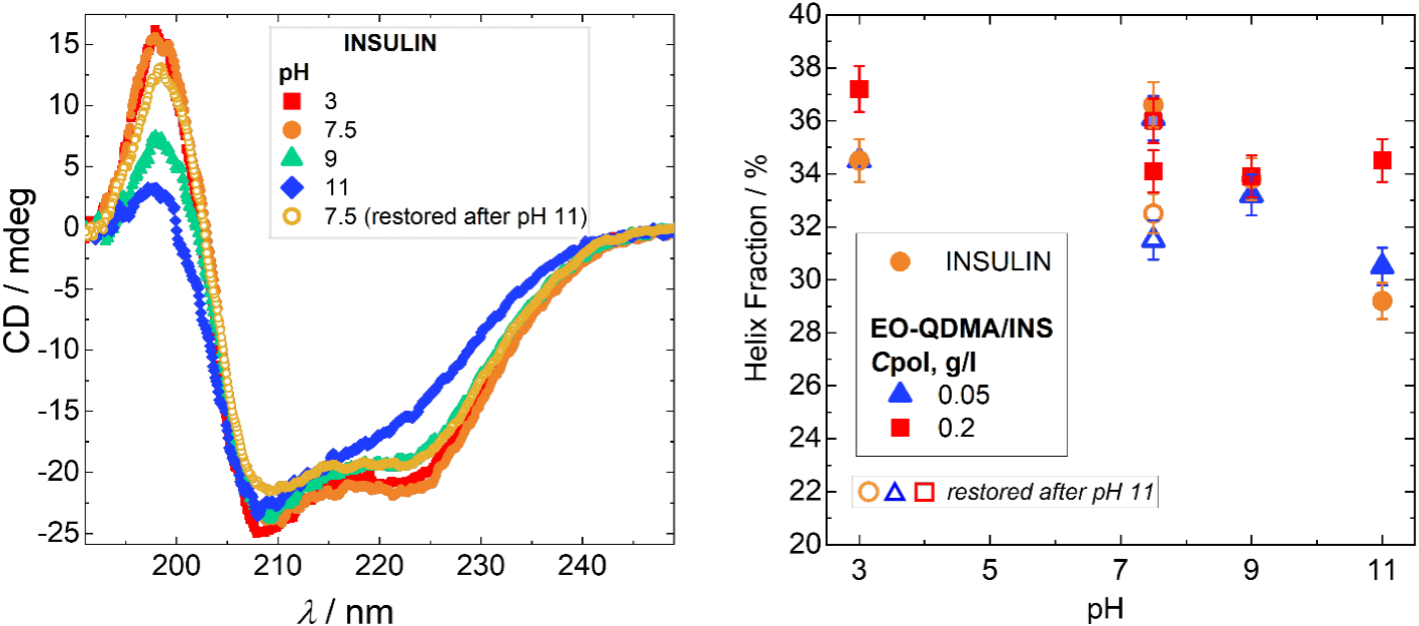
(Left) CD spectra of insulin at pH 3, 7.5, 9, and 11, insulin concentration 0.2 g/L, and temperature 25 °C. The samples were prepared at pH 7.5, and then the pH was adjusted to other values. The sample at pH 11 was then adjusted back to pH 7.5 to validate the reversibility of the protein. (right) Fraction of α-helices for insulin and insulin/EO-QDMA complexes as a function of pH. Open symbols correspond to helix fraction after pH 11 was titrated back to pH 7.5. The fractions of α-helices were obtained from the fitting of CD spectra using the BestSel method^31^ (see fitting curves and fractions of other secondary structure basis components in Figures S2 and S3 in the Supporting Information).

The pH-reversibility of secondary structure changes was verified by titrating insulin and EO-QDMA/insulin complexes from pH 7.5 to 11 and back to pH 7.5 (Figure 2, right, open symbols). The results prove that the secondary structure is mostly restored after the pH was increased to 11 and decreased to 7.5, but the fraction of helix structure decreased by 4–5 percentage points in comparison to initial values for both insulin and the EO-QDMA/insulin complex at low polymer concentration, while in the excess of polymer, the fraction of helix slightly increased (by 2 percentage points) that confirms the higher sensitivity of insulin to pH variation at low polymer concentrations.

### Temperature-Induced Conformation Transition of Insulin

Since insulin molecules unfold at a temperature of ca. 70 °C, significant changes should be observed when the protein is heated above this value. Figure 3 shows CD profiles of insulin heated from 25 to 80 °C with a heating rate of 1 °C/min. Between 70 and 75 °C, a clear transition from α-helices to β-sheets is observed by reduction of the peak intensity at 198 nm to zero and a significant decrease in the intensity of the peak at 223 nm. The BestSel fit confirms the reduction of the helices fraction by 16 percentage points and an increase of turns (by 3 percentage points), parallel β-sheets (by 3.5 percentage points), and other secondary structure components. However, the cooling of insulin down to 25 °C almost completely restored the number of α-helices and β-sheets as before heating, and the protein unfolded reversibly.

**Figure 3:**
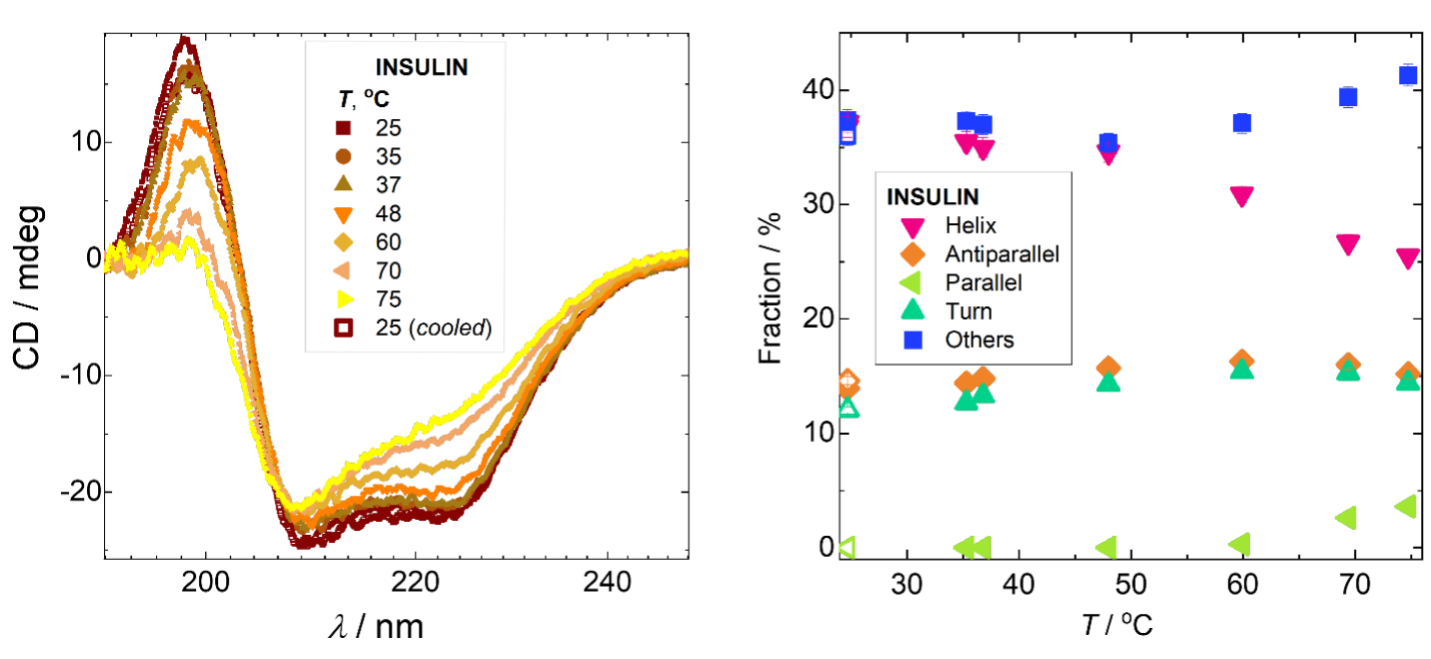
(Left) CD spectra of insulin at a concentration 0.2 g/L and pH 7.5, heated from 25 to 80 °C and further cooled to 25 °C. (Right) The fractions of α-helices, parallel and antiparallel β-sheets, turns, and other secondary structure basis components obtained from fitting CD spectra using the BestSel method^31^ (see fitting curves in Figure S2 in the Supporting Information). Open symbols at 25 °C correspond to values obtained after cooling the sample. The error bars for fractions did not exceed 3%.

Encapsulation of insulin enhances β-sheet formation even at low temperatures (see Figure 4, left) at high polymer content. Increasing temperature leads to protein unfolding, which is faster in the complexes in comparison to the protein in the bulk solution. At high polymer content, and therefore a higher fraction of insulin encapsulated, a clear transition is observed in the temperature range between 40 and 70 °C. Above the unfolding temperature, only 9% of helices remain for the complexes at high polymer concentration. This observation indicates that insulin molecules surrounded by polyelectrolytes are less stable under high temperature and have a stronger tendency to unfold in comparison to insulin in aqueous solution, which could be explained by the different oligomeric state of insulin. Indeed, it has been shown that the most thermodynamically stable form of insulin is a hexamer, while other oligomers are less stable and more biologically active.^8,33^ Hexamers have 3 conformational states (tense T6, relaxed R6, and T3R3) with hydrophobic patches inside the oligomer, which makes it more stable.^34^ Moreover, polyelectrolyte condensation onto the protein can trigger fibrillation at a lower temperature, due to the difference in “local pH” and pH in the bulk, as well as other specific interactions that act in favor of protein unfolding. The charge of weak ionizable groups in polyelectrolytes, peptides, and proteins, as well as their conformation, are strongly affected by the electrostatic interaction between ionizable groups.^35–38^

**Figure 4:**
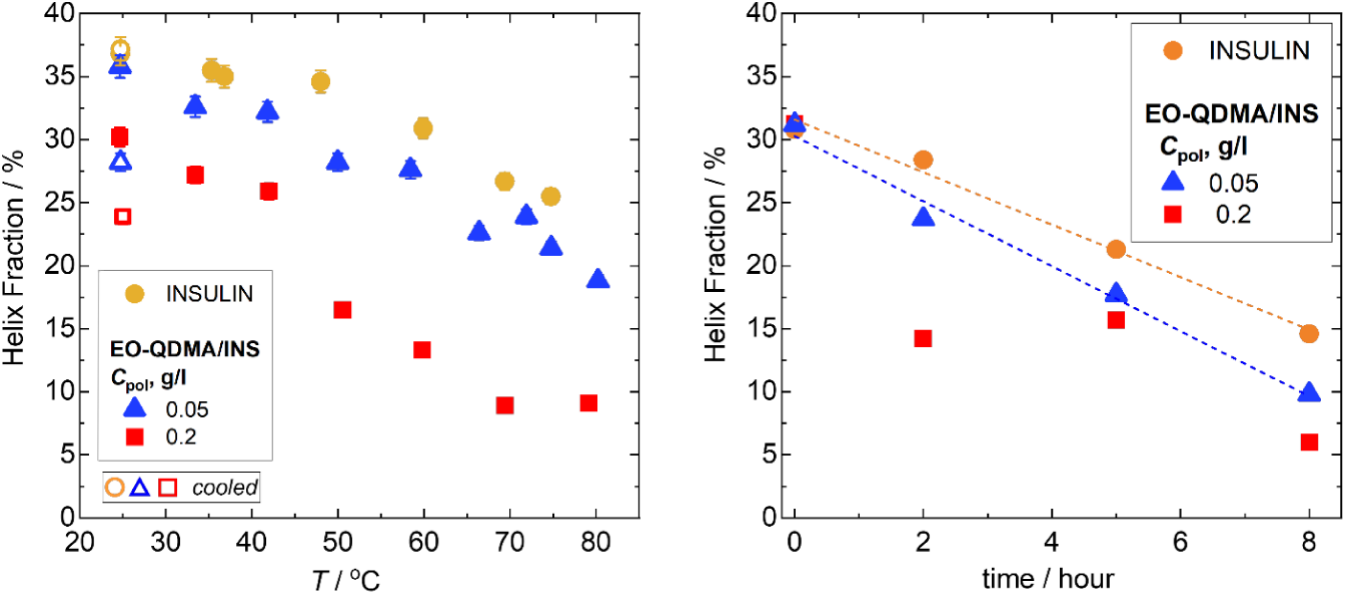
Fractions of α-helices for insulin and EO-QDMA/insulin complexes at polymer concentration 0.05 and 0.2 g/L obtained from fitting CD spectra using the BestSel method^31^ (see fitting curves and fractions of other secondary structure basis components in Figures S2, S4, and S5 in the Supporting Information): (left) temperature dependence; open symbols at 25 °C correspond to values obtained after cooling the sample, and (right) time dependence for samples heated at 80 °C for 0–8 h, followed by cooling down to 25 °C; the dashed lines are the linear fittings of experimental data. The error bars for helix fractions did not exceed 3%.

The bare insulin that was heated from 25 to 80 °C (heating rate 1 °C/min) and rapidly cooled down to 25 °C restores its initial secondary structure. In contrast, insulin in the polyelectrolyte environment manifests irreversible secondary structure changes (helix fraction decreases by 7 percentage points; Figure 4 left, open symbols). This observation confirms the lower thermal stability of encapsulated insulin.

### Thermal Stability of Insulin

To verify the reversibility of the bare insulin unfolding, we performed CD measurement for the samples heated for 2–8 h at 80 °C and measured their spectra after cooling to 25 °C (Figure 4 right). After 8 h of sample heating, the maximum at 198 nm has zero intensity, indicating the loss of helical structure. The results of the curve fitting show that the loss of helical structure in time is almost linear for insulin in the bulk and at low polymer concentration (see Figure 4, right). The addition of polymer to the system decreases the stability of the protein, and the unfolding becomes faster. At high polymer concentration, no linear behavior is observed anymore, and there is a sharp 2-fold decrease in the content of helices, which is reduced even further to 6%. The possible reason for the nonmonotonous behavior of insulin α-helix content in the presence of polyelectrolyte excess could be the combination of the cooperative nature of polyelectrolyte condensation and insulin oligomers reorganization within the nanoparticle core, leading to the change of insulin aggregation state.

To sum up, the encapsulation of insulin in polymer particles enhances the stability of the protein secondary structure under pH change but weakens the stability against high temperatures. This effect might be attributed to the different oligomeric states of insulin in bulk solution, where predominantly hexamers are formed, and in the complex, where hexamers and trimers coexist.^11^ The trimers within the complex are less stable and more sensitive to temperature gradients.

### Temperature-Induced Morphological Transition

In order to obtain information on insulin morphology in the nanoscale range, we performed SANS measurements with a temperature scan of insulin samples (Figure 5). At 25 °C, bare insulin forms hexamers with a radius of gyration 3.5 nm with a globular shape (see Figure 5a, c),^11^ and since the physiological pH is close to the isoelectric point of insulin, the protein is slightly negatively charged, so no structure factor is observed on SANS curves. The peak maxima in the Kratky plot (*q* ^2^ *I*(*q*) as a function of *q*, where *q* is the scattering vector and *I*(*q*) is the scattering intensity) at *q* = 0.08 Å^–1^ (Figure 5c and Table 1) could be used to estimate the pseudo-Guinier radius, *R*_pseudo_, of the insulin molecules, equal to $R_{\mathrm{pseudo}}=\sqrt{3}/q_{\max}$ , and at 25 °C, *R*_pseudo_ is 2.2 nm. The peak at *q* = 0.25 Å^–1^ is a form factor of a globular protein and indicates a folded insulin structure. Heating insulin up to 80 °C for 8 h leads to an irreversible partial unfolding of the protein (decrease in the peak height at *q* = 0.25 Å^–1^). The appearance of a correlation peak in the log–log SANS plot at 0.11 Å^–1^ indicates the arrangement of insulin molecules with a correlation distance of 5.7 nm after protein unfolding and packing into larger aggregates. In addition, no Guinier regime is observed, due to the particle size exceeding the available *q*-range. The slope of the SANS curve in double logarithmic scale is −3.8, indicating the nearly sharp interface of densely packed insulin aggregates. Cooling the insulin aggregates down to 25 °C does not recover insulin morphology, and insulin molecules remain in an aggregated unfolded state. It is noticeable that the second peak at 0.25 A^–1^ disappears after cooling because insulin fully loses the globular shape.

**Table 1:** Fitting Parameters for the Kratky Plot of SANS Curves of Bare Insulin and EO-QDMA/Insulin at pH 7.5, Fitted Using Bigaussian Function: Peak Center, *Q*_C_, Peak Height, *H*, and Width, *W*_1_ and *W*_2_.^39^,^a^

	Insulin				EO-QDMA/insulin			
*T* (°C)	*Q*_c_ (Å^–1^)	*H* (10^–4^)	*W*_1_ (Å^–1^)	*W*_2_ (Å^–1^)	*Q*_c_ (Å^–1^)	*H* (10^–4^)	*W*_1_ (Å^–1^)	*W*_2_ (Å^–1^)
**25**	0.08	24.6	0.06	0.07	0.01	20.2	0.005	0.009
	0.26	13.8	0.06	0.17	0.11	4.1	0.026	0.026
**80**	0.11	1.4	0.03	0.06	0.02	3.9	0.004	0.005
	0.25	0.7	0.11	0.03	0.10	3.3	0.053	0.063
**25 (cooled)**	0.11	1.8	0.03	0.08	0.02	3.1	0.004	0.004

a The error bars did not exceed 4%.

**Figure 5:**
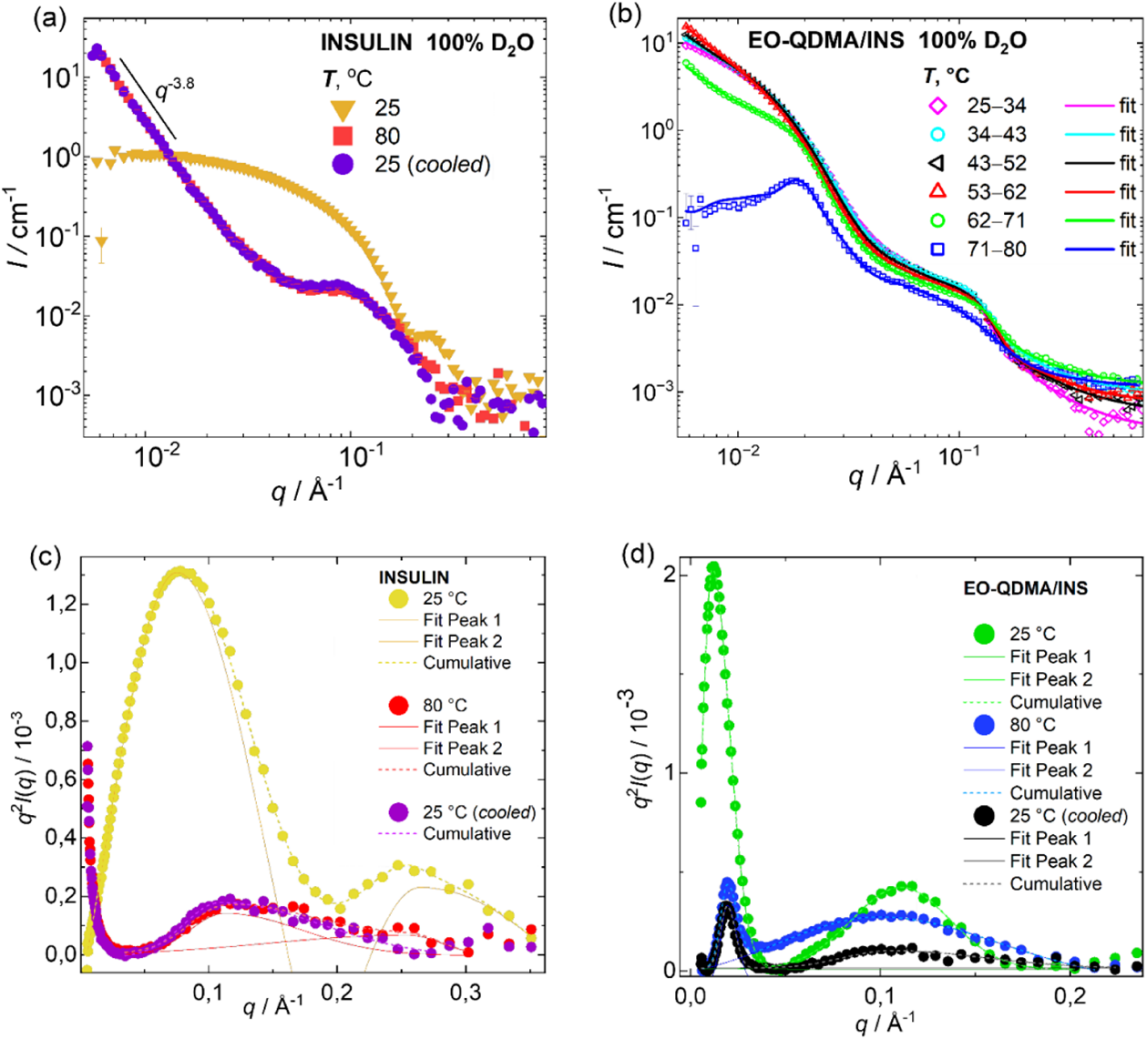
SANS data of (left) insulin and (right) EO-QDMA/insulin complexes measured at pH 7.5 at 25–80 °C and after cooling down to 25 °C. (a, b) SANS curves fitted using the form factors of Spherical Core with Gaussian Chains Attached,^24,25^ Broad Peak,^26^ and the structure factors of either Modified Caillé ^27,28^ or Mass fractal.^29,30^ The SANS curves with indicated temperature intervals were obtained by averaging the data collected over a 10 °C temperature window during continuous heating at a rate of 1 °C/min (see the contribution of each model and shifted curves with fitting in Figure S7 in the Supporting Information). (c, d) Kratky plot fitted using the Bigaussian function.^39^

The visual inspection of SANS curves for the EO-QDMA/insulin complexes shows that heating from 25 to 62 °C does not change the scattering profile in the high and mid-*q* range; however, the forward scattering intensity in the lower *q*-range increases with increasing temperature due to the enhanced interaction between particles, and therefore, a strong effect of structure factor on scattering curves. Between 62 and 71 °C, there is a transition from nanoparticle solution to the ordered microstructure (Figure 5b).

In our previous work, we have shown using cryogenic transmission electron microscopy (cryo-TEM), SAXS, and SANS with contrast variation that the EO-QDMA/insulin complexes form particles with a core–shell structure.^11^ The SANS curves for the complex were fitted using the form factor of a Sphere with Gaussian Chains attached^24,25^ because the model from one hand has the required topology of core–shell structure known from the preliminary experiments and contains the minimal required number of fitting parameters to describe the properties of the nanoparticles without significant ambiguity.^11^ This model allows us to determine such important parameters as the radius of the spherical core, the gyration radius of the Gaussian chains attached, the excess scattering lengths of a core and a shell, and the aggregation number.^24,25,40,41^ The theoretical excess scattering lengths were calculated using the SASfit software^23^ based on known chemical composition and mass densities. The Schulz–Zimm distribution^42,43^ was used to account for the polydispersity of a core radius. The correlation peak at *q* 0.1 Å^–1^ was fitted using the Broad Peak form factor to determine correlation distance and describe the local ordering inside a nanoparticle’s core.^26^ From the fitting results, we have concluded that a particle has a spherical core of radius 13 nm surrounded by a polymeric shell with a gyration radius of 3 nm with the polydispersity of the core radius 0.3.^11^ Using the contrast variation technique for SANS, we have unambiguously shown that the correlation peak at *q* = 0.1 Å^–1^ stems from the ordering of insulin oligomers in the core of the complex; the distance between the oligomers is equal to 6 nm and is kept due to the electrostatic repulsion between them.

In this work, the SANS scattering curves for the EO-QDMA/insulin complexes at elevated temperatures were analyzed by the same fitting model described above, in particular we used: (1) the Spherical Core with Gaussian Chains Attached model^24,25^ to describe the changes in core radius (*R*_a_), gyration radius of the polymer chains in the shell (*R*_g_), their excess scattering lengths (*r*_C_ and *r*_S_), aggregation number (*N*_agg_ was fixed to 100 based on previous fitting results); (2) Schulz–Zimm distribution for core radii (sigma is between 0.3 and 0.4); (3) Broad Peak model^26^ to fit the correlation peak at *q* ca. 0.08 Å^–1^ corresponding to the correlation distance between insulin oligomer within the core (fitting parameters: forward scattering of the broad peak, *I*_0_, correlation length, *x*_i_, peak position, *q*_0_, powers *p* and *m*). In addition, a structure factor is manifested at low *q* for elevated temperatures (Figure 5b). In light of two distinct patterns of the structure factor behavior as the function of temperature, we used different models to characterize the features of the scattering profiles: Mass Fractal model (Exp­(−*x* ^a^) Cut-Off)^29,30^ for the temperature interval 25–62 °C for the upturn of the scattering intensity and Modified Caillé structure factor^27,28^ for the temperature range 62–80 °C to describe the downturn of the scattering intensity together with the correlation peak at *q* 0.018 Å^–1^.

For the Mass Fractal model, the fitting parameters are the characteristic dimension of individual scattering objects (*r*_0_), the size of the fractal aggregate (*χ*), and fractal dimension (*D*).^29,30^ The fractal dimension is correlated to the slope of the curve at the lowest *q* that can be used to describe the aggregates’ compactness (aggregates are more compact at a steeper slope); however, this model cannot be used for more detailed analysis due to the lack of data points at lower *q*. For the Modified Caillé structure factor, the fitting parameters are the number of layers in the stack (*N*_LR_), stacking separation (*d*), and Caillé parameter (*η*_Caillé_) (Figure 5b and Table 2).^27,28^

**Table 2:** Fitting Parameters of Temperature-Dependent SANS Curves of EO-QDMA/Insulin complexes, Obtained Using the Sphere with Gaussian Chains Attached Model,^29,30^ Broad Peak,^26^ and Modified Caillé ^27,28^ or Mass Fractal^29,30^ Structure Factors.^a^

	Form Factor^b^		Mass Fractal			Broad Peak				
*T* (°C)	*R*_a_ (nm)	*R*_g_ (nm)	*r*_0_ (nm)	*χ* (nm)	*D*	*I*_0_ (cm^–1^)	*x*_i_ (Å)	*q*_0_ (Å^–1^)	*m*	*p*
25–34	10.8	3.8	27.8	–	1.76	0.009	24.9	0.08	5.1	0.6
34–43	11.4	3.5	11.8	>q.r.^c^	2.95	0.009	22.0	0.08	4.8	0.8
43–52	11.4	3.5	10.7	>q.r.^c^	2.96	0.009	9.9	0.07	3.7	5.0
53–62	12.1	3.5	13.0	>q.r.^c^	2.84	0.008	7.0	0.06	3.9	10.0

			Modified Caillé							
			*N*_LR_	*d* (Å)	*η*_Caillé_					
62–71	12.5	3.1	2.2	260	1.57	0.005	14.0	0.07	5.3	1.4
71–80	8.6	1.0	3.3	304	0.96	0.011	6.2	0.04	2.0	5.0

a The fitting parameters are a core radius (*R*_a_), gyration radius of the polymer chains in the shell (*R*_g_), the forward scattering of the broad peak (*I*_0_), correlation length (*x*_i_), peak position (*q*_0_), Powers *p* and *m*, the characteristic dimension of individual scattering objects (*r*_0_), the size of the fractal aggregate (*χ*), fractal dimension (*D*), the number of layers in a stack, (*N*_LR_), stacking separation (*d*), and Caillé parameter (*η*_Caillé_). Excess scattering lengths of a core and the shell were fixed to *r*_S_ 3.9 Å and *r*_C_ 7.5 Å.

b Sphere with Gaussian cains attached.^24,25^

c Fitted value is outside experimental data points; to calculate the dimension of the mass-fractal aggregate, χ, ultra-SANS experiments at lower *q* are required.

At moderate temperatures, the core size slightly increases, while the shell thickness (*R*_g_) remains the same that is arguably caused by a partial α-helices to β-sheets transition and rearrangement of the insulin oligomers within the core of the nanoparticles that is also in line with the shift of the correlation peak from 0.08 to 0.06 Å^–1^ due to the increased distance between insulin oligomers. The upturn at low *q*, appearing at moderately elevated temperatures, indicates the enhanced interparticle interaction and their aggregation. The dimensions of individual scattering objects (*r*_0_) obtained from the Mass fractal contribution match the size of EO-QDMA/insulin nanoparticles *R*_a_ (Table 2), suggesting that the fractal objects are composed of EO-QDMA/insulin nanoparticles. The density of EO-QDMA/insulin aggregates does not show any significant changes, as evidenced by the fractal dimension, *D*, value (Table 2). The only changes that are visible for the temperature range of 25–62 °C occur with insulin oligomers inside a nanoparticle core. SANS results provide additional insight into the spatial rearrangements of insulin oligomers; the correlation length (*x*_i_) drops significantly with increasing temperature. We conclude that the temperature rearrangement of insulin oligomers results in more ordered structures with a faster decay of the correlation length due to further distance from each other. The heating of the complexes above the unfolding temperature (>70 °C) causes irreversible changes in the complex morphology. The structure factor shows a completely different pattern at low *q* (Figure 5b). The additional ordering of the complexes into multilayered structures is confirmed by the appearance of a correlation peak at *q* 0.018 Å^–1^ that corresponds to the periodic distance of 350 Å (35 nm) that roughly equals the nanoparticle diameter (2*R*_a_ + 2*R*_g_ = 2 × 12.5 nm + 2 × 3.1 nm = 31.2 nm). Given that fact and the assumption that the increased fraction of β-sheets will favor the formation of layers, we used the Modified Caillé structure factor to describe these additional structure features at low *q*, which describes multilamellar structures with bending fluctuation disorder. The structure factor allows us to determine the number of layers in stack, *N*_LR_, stacking separation, *d*, and Caillé parameter, *η*_Caillé_.^27,28^ The fitting results imply the existence in solution of semiflexible multilayered structures composed of 2–3 layers with an interlayer distance of 300 Å (Table 2).

The contrast variation SANS measurements allow us to mask the scattering from the QDMA block at a 70/30 D_2_O/H_2_O ratio or insulin at a 35/65 D_2_O/H_2_O (see Figure 6). This becomes possible due to the significant difference in scattering length densities of the EO block, QDMA block, and insulin for neutrons. The experiment shows that after heating, both correlation peaks at 0.1 and 0.01 Å^–1^ disappear, indicating that they both have an origin of insulin arrangement within the complex and agglomerates. At 35% D_2_O and 80 °C, the scattering from single polymer chains of EO-QDMA copolymer is observed. The global fitting of the SANS curves with Core with Gaussian Chains Attached, Broad Peak models, and Modified Caillé structure factor for the complexes at the same temperature but different contrast conditions was performed to ensure the validity of the selected fitting model (Figure 6, Table 3). As one can see from Figure 6, the different sets of data for 100, 70, and 35% of D_2_O were successfully fitted, giving strong evidence that the selected fitting model is correct. The fitting error did not exceed 5% for all fitting parameters.

**Figure 6:**
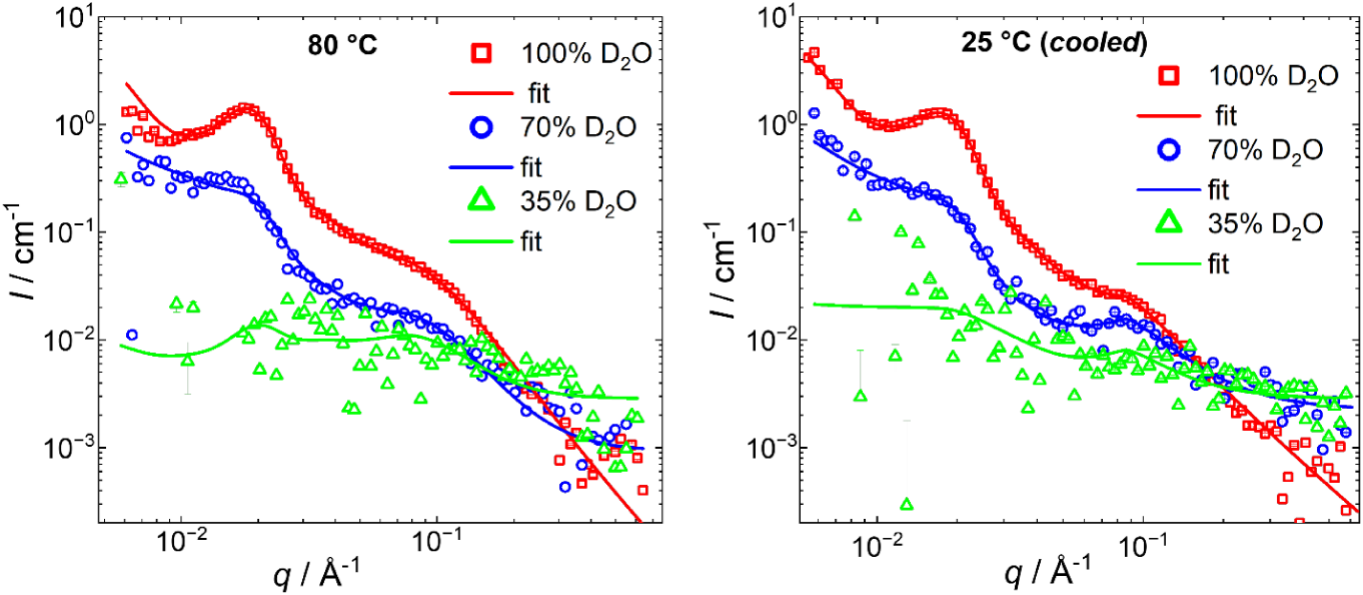
Contrast matching for SANS of EO-QDMA/insulin complexes at pH 7.5 (left), heated to 80 °C, and (right) cooled after 8 h of heating, measured in 100, 70, and 35% of D_2_O. The curves were fitted using the form factor of the Spherical Core with Gaussian Chains Attached model, Broad Peak, and Modified Caillé structure factor. The incorporation of the Modified Caillé structure factor into the fitting model brings additional ambiguity to the fitting routine, having 14 fitting parameters, 3 of which are fixed. To verify our previous findings, the contrast variation SANS measurements, followed by the global fitting of the experimental data for different contrasts, were performed.

**Table 3:** Fitting Parameters for SANS Curves of EO-QDMA/INS Measured at 25 °C, 80 °C, and 25 °C after Cooling (25 °C), in 100, 70, and 35% D_2_O, and Obtained by Global Fitting Using the Spherical Core with Gaussian Chain Attached model,^24,25^ Broad Peak Form factor,^26^ and Modified Caillé ^27,28^ Structure Factor.^a^

*T* °C	D_2_O%	*R*_a_ nm	*R*_g_ nm	*r*_C_ Å	*r*_S_ Å	*N*_LR_	*d,* Å	η_Caillé_	*I*_0_ cm^–1^	*x*_i_ Å	*q*_0_ Å^–1^	*m*	*p*
25	100	12.8	3.3	7.5	3.9	–			0.04	7	0.07	3.2	12.4
	70			–3.3	–1.9				0.02				
	35			–1.6	5.3				0.01				
80	100	8.2	4.5	7.5	3.9	2.3	296	0.55	0.03	13	0.08	1.7	1.6
	70			–3.3	–1.9				0.01				
	35			–1.6	5.3				0.01				
25c	100	10.7	4.2	7.5	3.9		293	0.64	0.04	16	0.09	1.3	1.5
	70			–3.3	–1.9				0.02				
	35			–1.6	5.3				0.01				

a The fitting parameters were the radius of the Core, *R*_a_, aggregation number, *N*_agg_, gyration radius of a polymer chain in a shell, *R*_g_, number of layers in stack, *N*_LR_, stacking separation, *d*, Caillé parameter, *η*_Caillé_, forward scattering of the broad peak, *I*_0_, correlation length, *x*_i_, peak position *q*_0_, powers *p* and *m*. The excess scattering length of the block, *r*_C_, and of the core, *r*_S_, were calculated based on known composition and mass densities and fixed as is in the fitting routine. For the core composition, the scattering of insulin was added based on our previously published data.^11^

The fitting revealed that the radius of the core slightly decreases due to the more ordered arrangement of insulin after unfolding, while the *R*_g_ of polymer chains slightly increases due to the decrease in surface area of the core, leading to a smaller space for polymer chains on the top of the core, and it forces the EO block to stretch (Table 3). After heating, there are 2 layers in the stack with a stacking separation of 26 nm, which corresponds to the diameter of the core/shell nanoparticles. This confirms the additional ordering of the nanoparticles within agglomerates. The peak position slightly shifts to higher *q* values due to the differences in the size of insulin multimers and the correlation distance between them after heating from 9 to 7 nm.

It is worth mentioning that polymer and protein interaction can be significantly affected by changing the solvent from H_2_O and D_2_O due to the different intra- and intermolecular hydrogen bonding between polymer, protein, and solvent. In our previous work, we observed that nanoparticles tend to form loose networks in D_2_O, possibly due to the stronger hydrogen bonding between polymer and protein molecules and weaker bonding with solvent molecules.^11^ However, using small-angle X-ray scattering (SAXS), measured in D_2_O and H_2_O, we demonstrated that SAXS profiles of the nanoparticles in the mid- and high-*q* range are not affected by changing the solvent. Therefore, using D_2_O for SANS experiments does not affect the fitting results.

### Microstructure of the Aggregates

The microstructure of the complexes after heating at 80 °C for 8 h was visualized with scanning electron microscopy (SEM, Figure 7). Even though the arrangement of insulin molecules is observed in SANS, on a microscale they form irregularly shaped large aggregates interconnected with each other, and no other long-distance correlation in the structure is observed. This confirms that the only trigger for unfolded insulin arrangement is short-range electrostatic repulsion between like-charged molecules. On the contrary, EO-QDMA/INS complexes form spherical-like aggregates with a size of 0.7–1 μm, consisting of highly arranged nanoparticles with encapsulated ordered insulin. These spherical aggregates of EO-QDMA/INS are further interconnected into larger particles. Thus, insulin loading into the polymer nanoparticles prevents insulin aggregation, since the protein remains isolated in the polyelectrolyte core, but does not prevent irreversible insulin unfolding within protein trimers.

**Figure 7:**
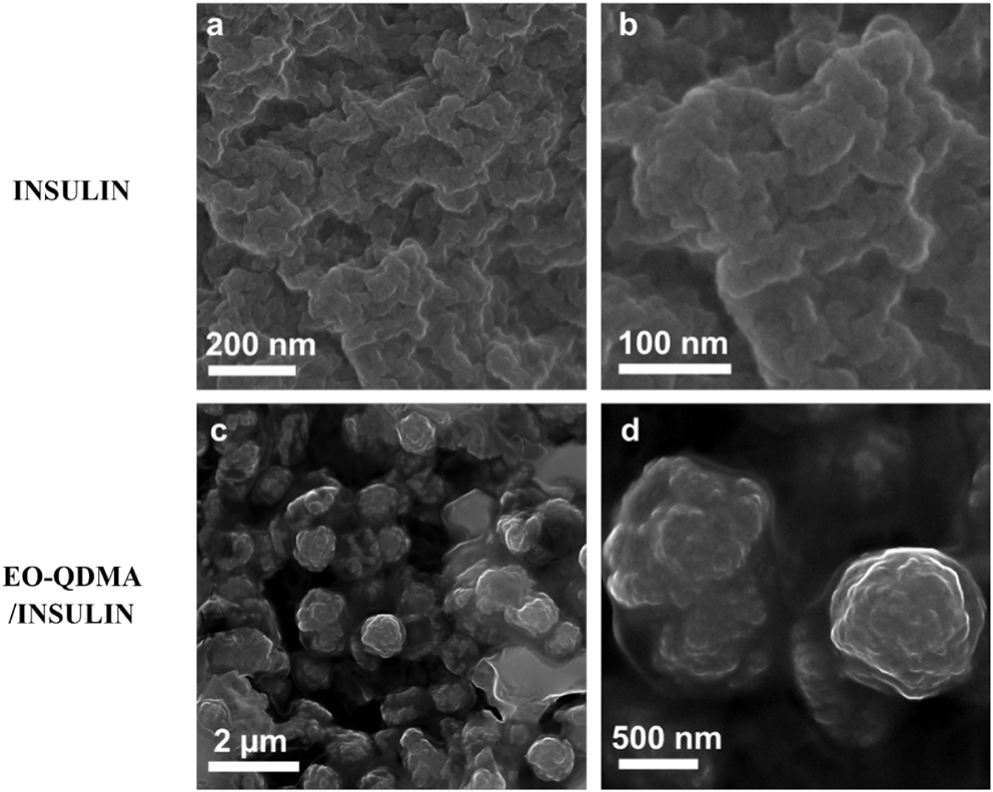
SEM images of (a, b) insulin and (c, d) EO-QDMA/INS in 50 mM NaCl complexes cooled to 25 °C after heating to 80 °C for 8 h.

## Conclusions

We characterized insulin stability and conformational changes under charge variation of the protein by pH variation and polycation condensation. Insulin stability in basic pH can be enhanced by encapsulation into polymeric nanoparticles and protein charge neutralization. However, the temperature sensitivity of the protein is enhanced in the polyelectrolyte environment due to the stronger hydrophobic interactions and different oligomeric state of the protein. The unfolding temperature of insulin is shifted from 75 °C for insulin in bulk solution (hexamers) to 50 °C within polymeric nanoparticles (trimers). Moreover, unfolded insulin molecules aggregate with short-distance ordering (correlation distance 6 nm), while encapsulated insulin molecules have both short-distance (6 nm) and long-distance (31 nm) ordering due to the formation of larger agglomerates from arranged polymer/insulin nanoparticles. The unfolding and agglomeration for both insulin and polymer/insulin complexes become irreversible after prolonged heating above denaturation temperature since it causes almost complete transition from α-helical to compact β-sheet structure (Figure 8). Therefore, these observations allow us to determine the effect of protein charge regulation on conformation and aggregation, which could be used for enhancing protein stability or the development of new smart protein-based biomaterials with controlled properties.

**Figure 8:**
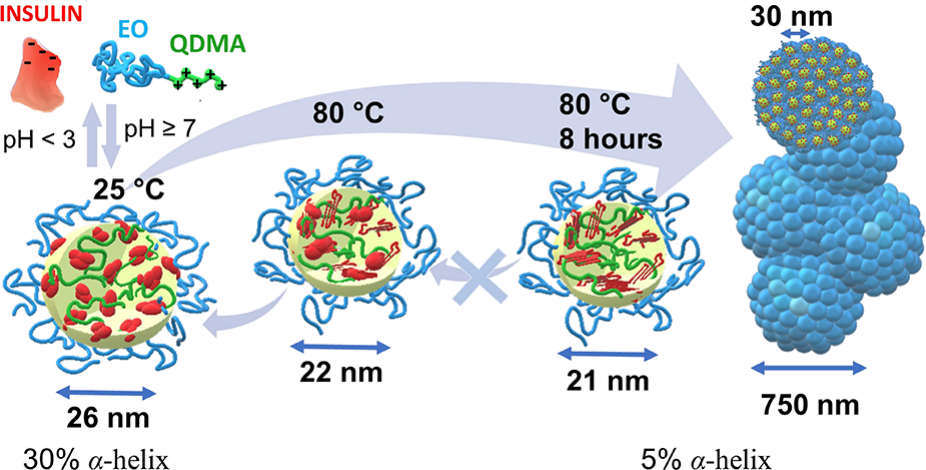
Evolution of EO-QDMA/insulin morphology under heating: at 25 °C, EO-QDMA/insulin nanoparticle has a spherical core consisting of polycation and globular insulin trimers, surrounded by a hydrophilic EO shell; after heating to 80 °C, part of insulin α-helices reversibly unfold to β-sheets within the complex; long-lasting heating leads to complete irreversible unfolding of the insulin and nanoparticle aggregation into spherical microparticles.

## Supplementary Material



## Notes

CD spectra, smoothed and fitting curves for CD data, fitting of SANS curves, and SEM images (PDF)
