# Supplementary material for: Encapsulated Control: Shaping Insulin Fibrillation through Polymer Confinement
Source: Biomacromolecules. 2025 Dec 24;27(1):845–54. doi: 10.1021/acs.biomac.5c02110 (PMC12801316; doi:10.1021/acs.biomac.5c02110)
Supplement: Supplementary file 1 [file bm5c02110_si_001.pdf]

SUPPORTING INFORMATION

Encapsulated Control: Shaping Insulin Fibrillation Through Polymer Confinement

Anastasiia Murmiliuk[1,2]*, Sergey K. Filippov[3], Hiroki Iwase[4], Kuno Schwärzer[5], Jürgen Allgaier[5], Aurel Radulescu[2]

[1] Department of Medical Biotechnology and Translational Medicine, Università degli Studi di Milano, Italy

[2] Jülich Centre for Neutron Science (JCNS) at Heinz Maier-Leibnitz Zentrum (MLZ), Forschungszentrum Jülich GmbH, Lichtenbergstraße 1, 85747 Garching, Germany

[3]DWI – Leibniz Institute for Interactive Materials, Forckenbeckstr. 50, 52056 Aachen, Germany

[4]Neutron Science and Technology Center, Comprehensive Research Organization for Science and Society (CROSS), Tokai, Ibaraki 319-1106, Japan

[5]Jülich Centre for Neutron Science (JCNS-1), Forschungszentrum Jülich, Leo Brandt Straße, 52425 Jülich, Germany

**E-mail: anastasiia.murmiliuk@gmail.com*

Content: Page

1. Circular Dichroism (CD) spectra S2
2. Small-Angle Neutron Scattering (SANS) curves S6
3. Scanning Electron Microscopy (SEM) images S8

1. Circular Dichroism (CD) spectra

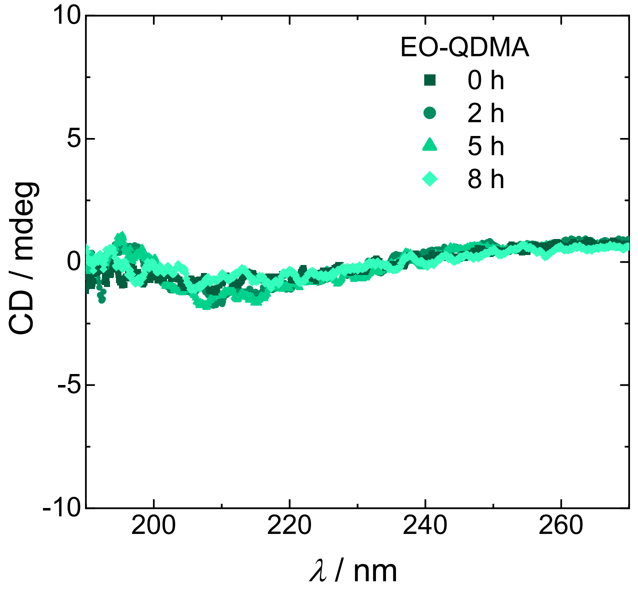

Figure S1. CD spectra of EO-QDMA copolymer at 25 °C and polymer concentration 0.2 g/l after heating the samples at 80 °C for 0–8 hours.

No CD signal is observed for polymer due to the low absorbance in UV-vis region.

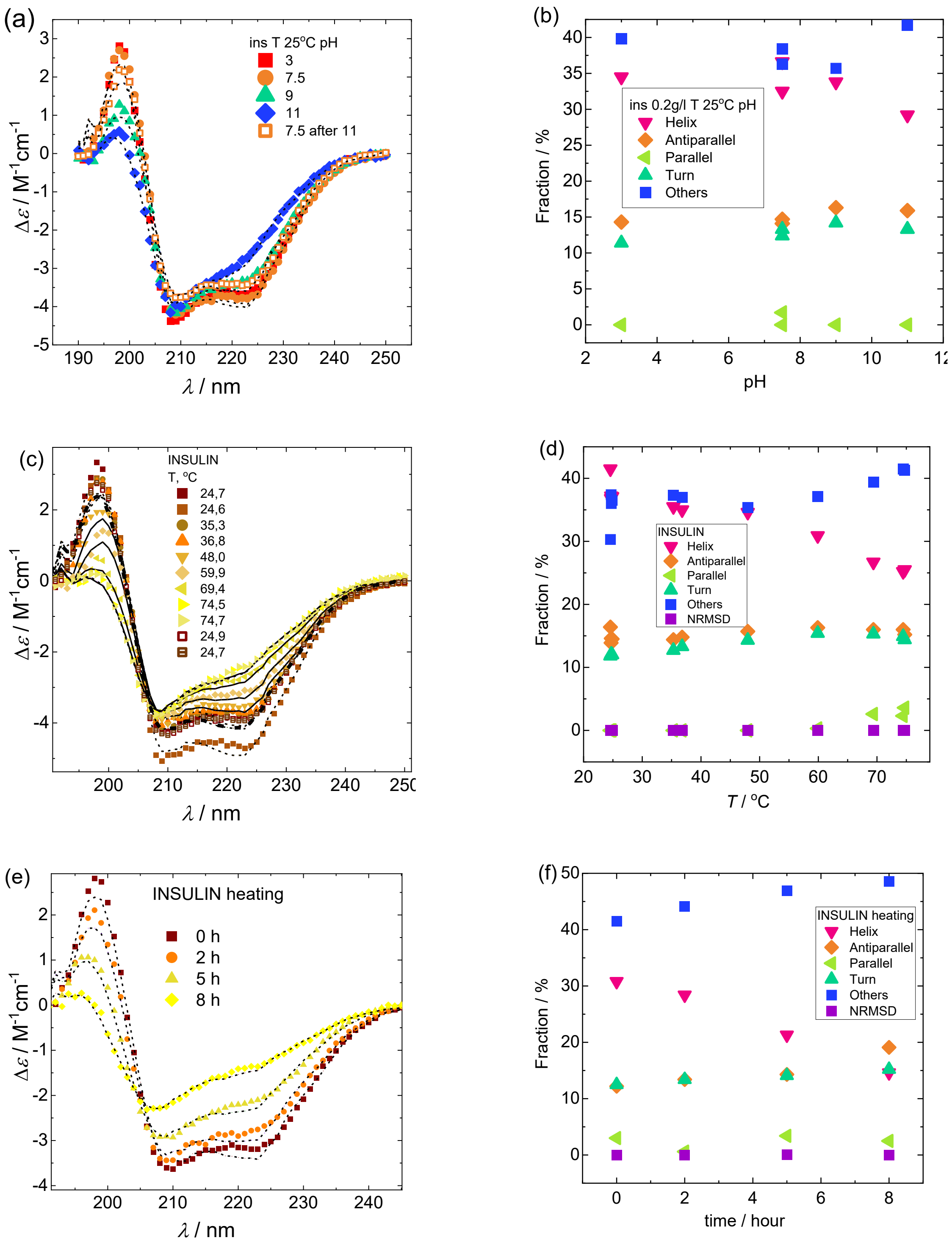

Figure S2. (left) CD spectra with fitting curves and (right) fractions of secondary structure basis components obtained from fitting CD spectra using BestSel method for insulin (a, b) at 25 °C at various pH, (c, d) in temperature range 25–75 °C and (e, f) at 25 °C after heating for 0-8 hours at 80 °C.

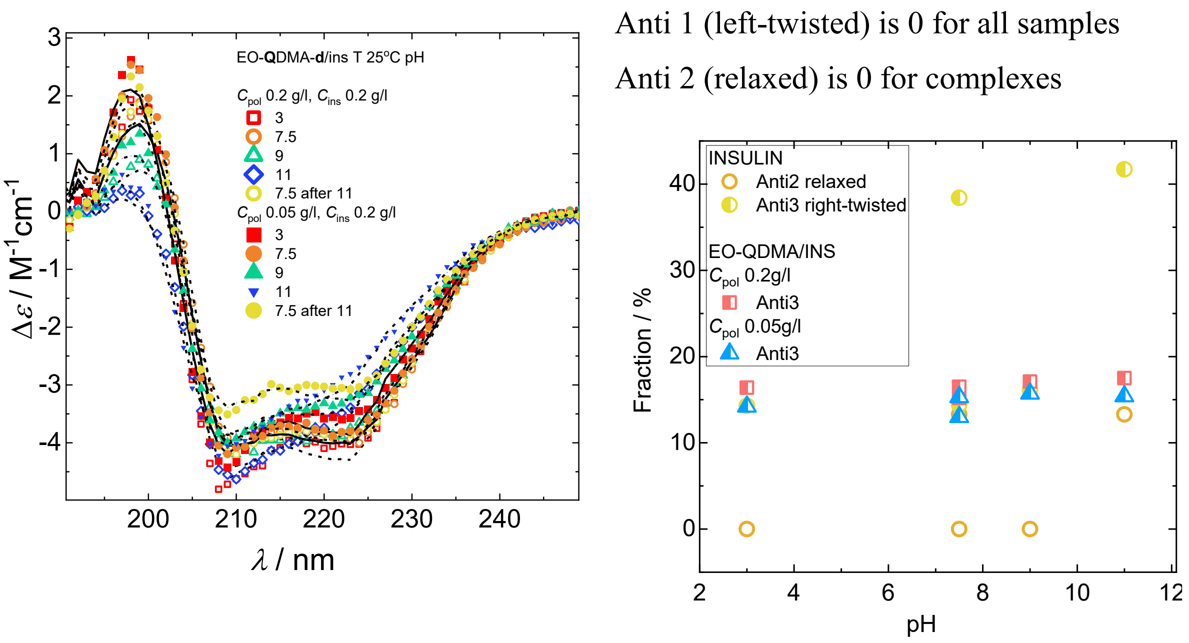

Figure S3. (left) CD spectra with fitting curves and (right) fractions of anti-parallel secondary structure basis components obtained from fitting CD spectra using BestSel method for insulin at 25 °C at various pH.

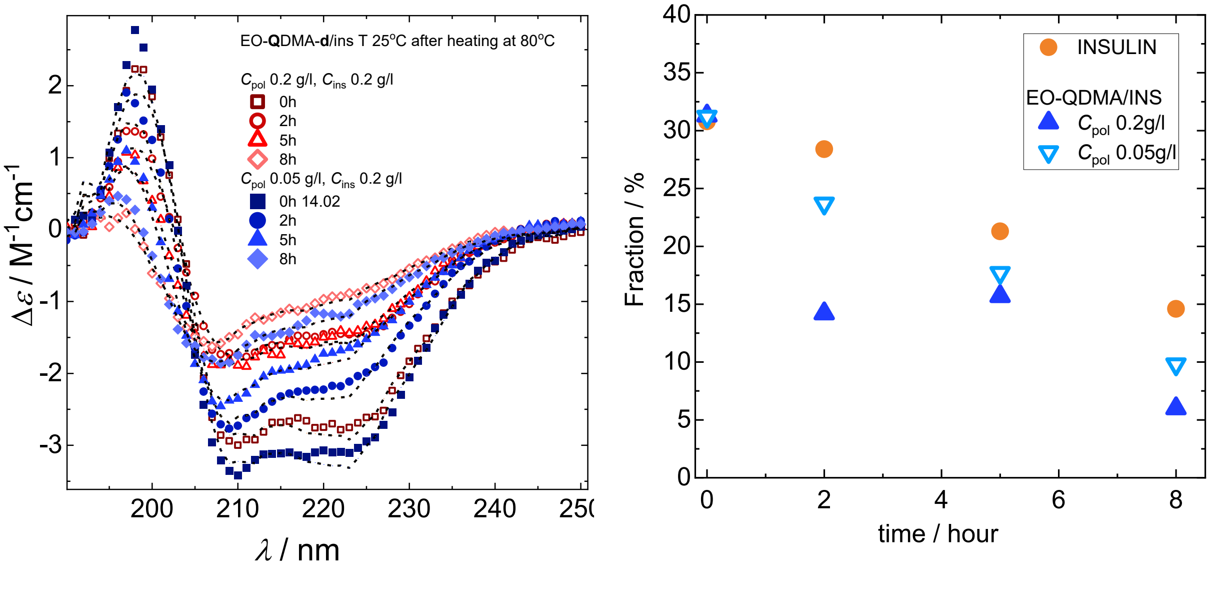

Figure S4. (left) CD spectra with fitting curves and (right) fractions of α-helices secondary structure basis components obtained from fitting CD spectra using BestSel method for insulin at 25 °C after heating for 0–8 hours at 80 °C.

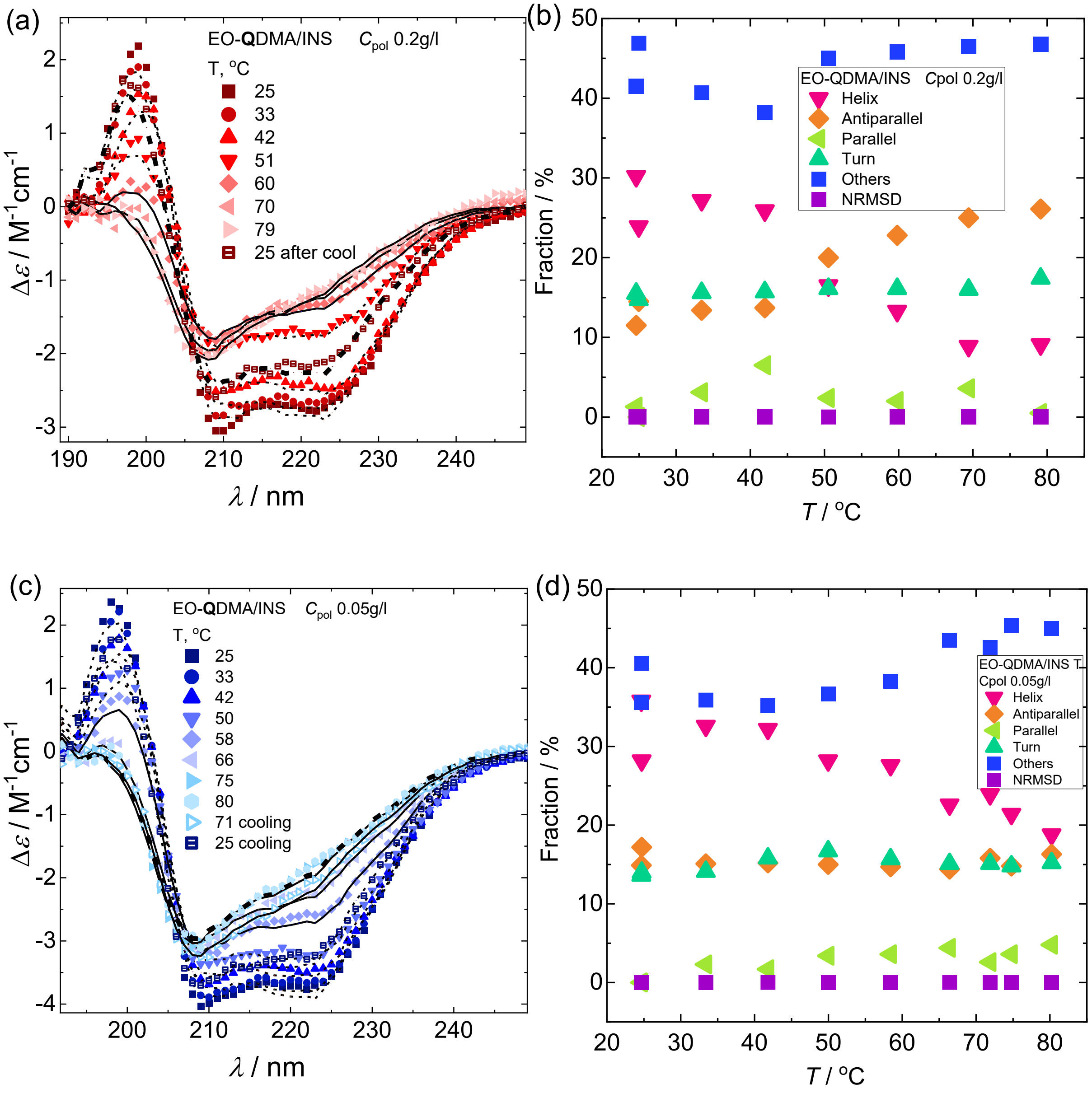

Figure S5. (left) CD spectra with fitting curves and (right) fractions of secondary structure basis components obtained from fitting CD spectra using BestSel method for insulin in temperature range 25–75 °C at pH 7.5 and polymer concentration (a, b) 0.2 g/l and (c, d) 0.05 g/l.

2. Small-angle neutron scattering (SANS) curves

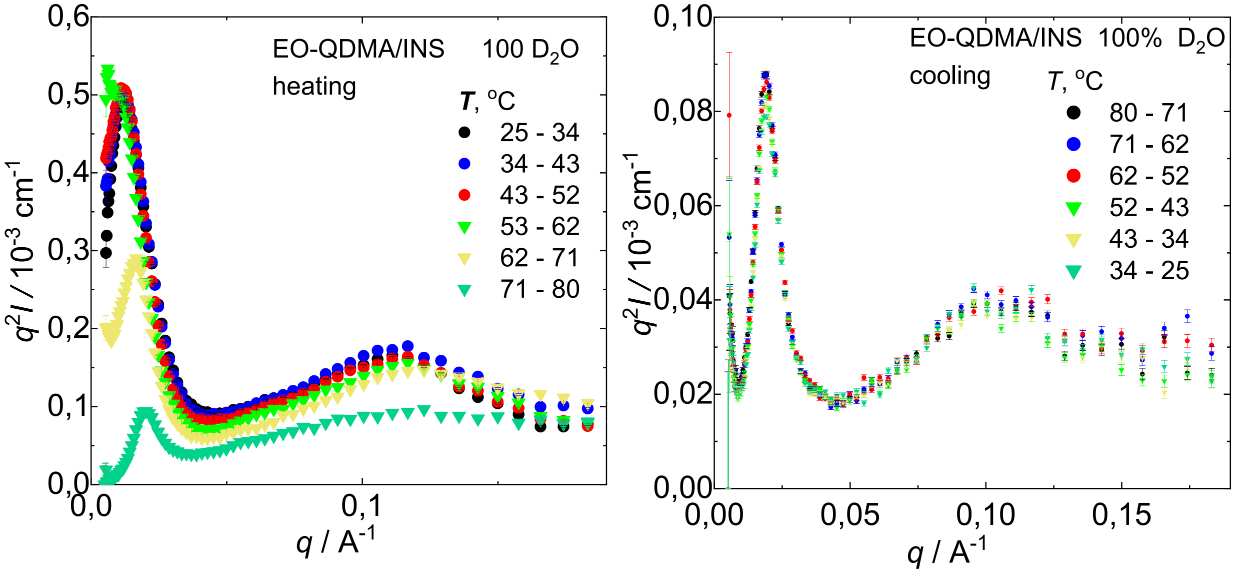

Figure S6. Kratky plot for (left) heating and (right) cooling EO-QDMA/INS complexes between 25 and 80 °C in 100 % D_2O.

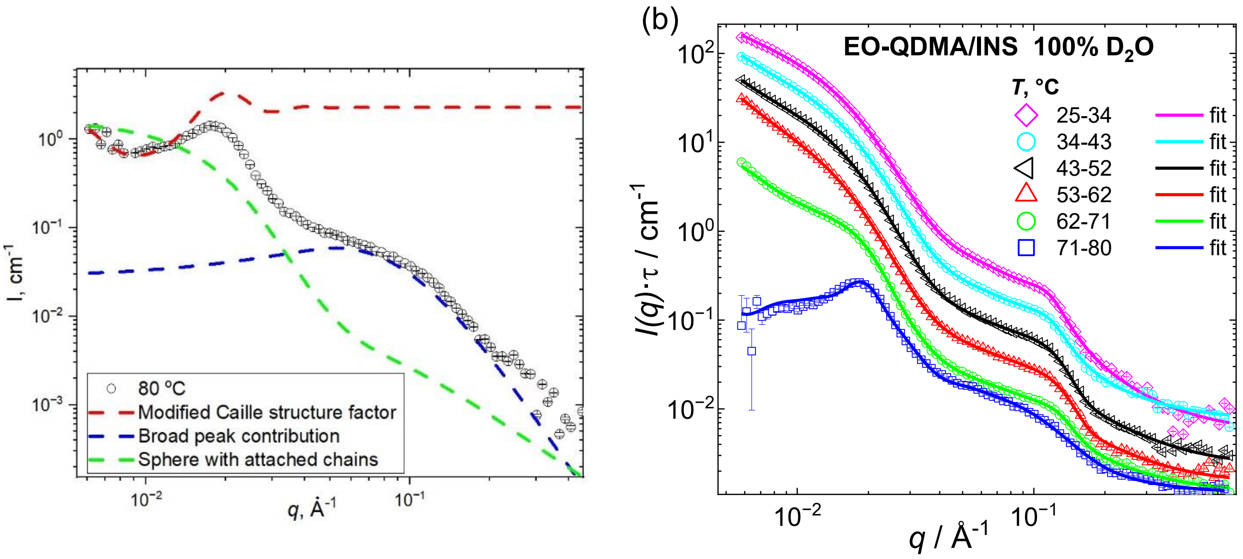

Figure S7. (a) Fitting of SANS curve for EO-QDMA/INS at 80 °C in 100 % D_2O using Sphere with Gaussian Chains Attached model, Broad Peak model and Modified Caille structure factor, and the contribution of each model to the resulting fitting. (b) Fitting of SANS curve for EO-QDMA/INS at 80 °C in 100 % D_2O using Sphere with Gaussian Chains Attached model, Broad Peak model and Modified Caille structure factor. The curves are shifted for better

observation of curves' profiles by the factor τ equal to 1 for the curve 71–80 °C and 62–71°C, τ =2 for 53–62 °C, τ =4 for 43–52 °C, τ =8 for 34–43°C, τ =16 for 25–34 °C.

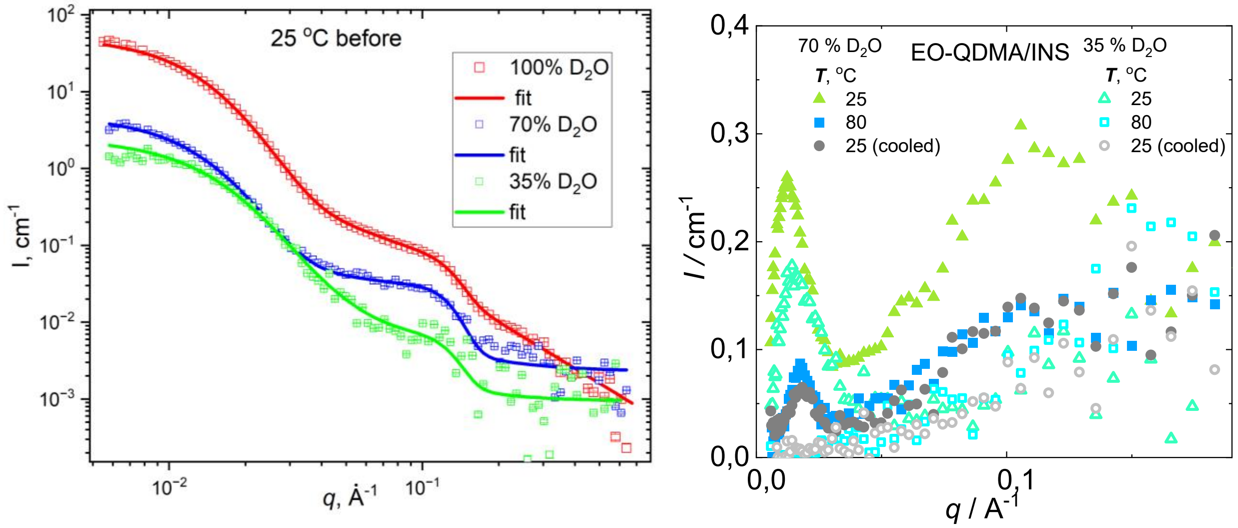

Figure S8. (left) SANS curves for EO-QDMA/INS complexes at 25 °C with contrast matching, measured at 100, 70 and 35 % D_2O and fitted using Sphere with Gaussian Chains attached model and Broad Peak model. (right) Kratky plot for SANS curves of EO-QDMA/INS complexes in 70 and 35 % D_2O measured at 25 °C, 80 °C and 25 °C after cooling.

3. Scanning Electron Microscopy (SEM) images

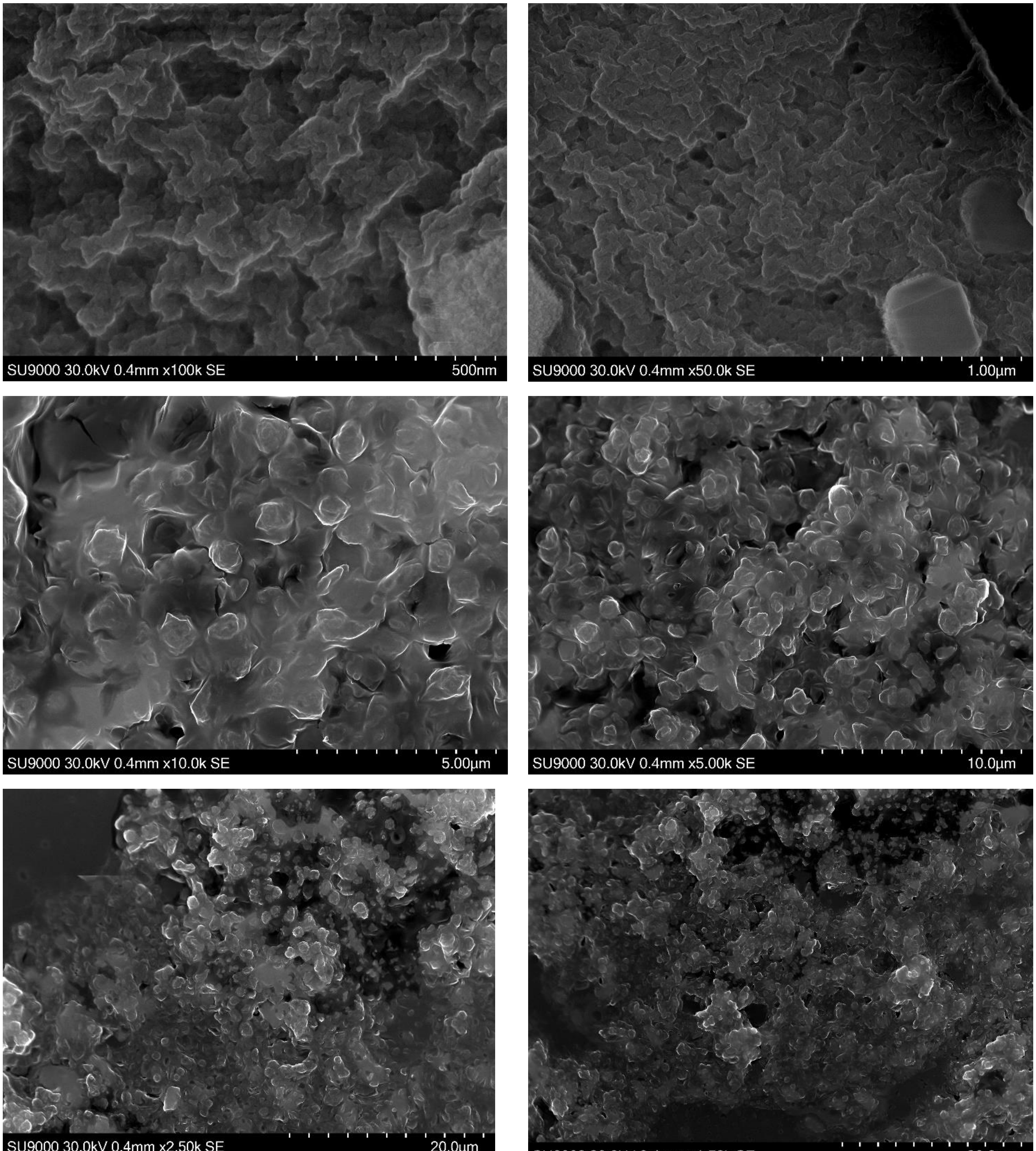

Fig. S9. Additional SEM images measured after heating the samples at 80 °C for 8 hours and cooling down to 25 °C for: (a,b) insulin at concentration 20 g/l, (b) EO-QDMA/INS at insulin concentration 20g/l and EO-QDMA concentration 5 g/l.
